# Progress towards prevention of suicide in India by improving print media reporting of suicide news: a repeat content analysis study in Tamil Nadu

**DOI:** 10.1136/bmjopen-2024-092652

**Published:** 2025-05-16

**Authors:** Gregory Armstrong, Tilahun Haregu, Mala Jayaseelan, Thomas Niederkrotenthaler, Anish Cherian, Vikas Menon, Vikas Arya, Lakshmi Vijayakumar

**Affiliations:** 1 Centre for Mental Health and Community Wellbeing, The University of Melbourne, Melbourne, Victoria, Australia; 2 Department of Psychiatry, Voluntary Health Services, Chennai, India; 3 Centre for Public Health, Medical University of Vienna, Wien, Austria; 4 Department of Psychiatric Social Work, National Institute of Mental Health and Neurosciences, Bangalore, India; 5 Department of Psychiatry, Jawaharlal Institute of Postgraduate Medical Education and Research (JIPMER), Puducherry, India; 6 Department of Psychiatry, The Voluntary Health Services Hospital, Chennai, Tamil Nadu, India; 7 SNEHA Suicide Prevention Centre, Chennai, India

**Keywords:** Suicide & self-harm, MENTAL HEALTH, PSYCHIATRY, India

## Abstract

**Abstract:**

**Objectives:**

Suicide rates in India are among the highest in the world, with the most recent suicide death rate estimates ranging between 18 and 21 deaths per 100 000 population (compared with the global average of 11/100 000). Responsible media reporting of suicide is one of the few evidence-based population-level suicide prevention interventions. Reports of recent suicides are a routine daily feature in major newspapers in India, and the reporting style carries many concerning features. In 2019, the Press Council of India adopted the WHO media guidelines, yet there has been no investigation as to whether this guidance is being followed. The aim of this paper was to systematically investigate whether the quality of print media reports of suicides has changed since the adoption of media guidelines for suicide reporting in India.

**Design:**

We used content analysis to assess the quality of suicide reporting against WHO guidelines in nine of the most highly read daily newspapers in the southern state of Tamil Nadu between June and December 2016 and June and December 2023. Our analyses of changes in reporting were based on a sample of 1681 print newspaper articles from 2016 and 512 print newspaper articles from 2023. Two-tailed t-tests and proportion tests on aggregate means and frequencies assessed whether the reporting characteristics had changed between 2016 and 2023.

**Results:**

There were small yet statistically discernible reductions in the proportion of articles containing various potentially harmful reporting characteristics, such as articles placed on the front page (4.9–1.8%, p=0.002) and articles mentioning the suicide method (92.7–86.5%, p<0.001). There were statistically discernible increases in the proportion of articles containing various potentially helpful reporting characteristics, such as recognition of the link between suicide and poor mental health (7.6–10.5%, p=0.035), mentions of suicide prevention support services/programmes (3.6–11.7%, p<0.001) and the provision of contact details for a suicide support service (2.5–8.8%, p<0.001). There was no statistically discernible improvement in several quality indicators, for example, providing a detailed account of the suicide method (43.1–38.9%, p=0.092), the naming of publicly accessible sites where suicides have occurred (8.2–10.0%, p=0.216), dispelling of suicide myths (2.0–1.8%, p=0.705) and drawing on expert opinions from mental health professionals (1.2–2.0%, p=0.238). In some instances, quality indicators had worsened, such as an increase in articles published in the first three pages of the newspaper (16.8–19.1%, p<0.001) and the use of monocausal explanations for the suicidal behaviour (53.4–70.7%, p<0.001). Analyses at the newspaper level showed that the small improvements that were observed were mainly driven by quite profound improvements in the quality of reporting by two English-language newspapers. For example, at The Hindu, there was a very large decrease in the proportion of articles mentioning the suicide method (85.7–14.3%, p<0.001) and increases in the proportion of articles dispelling suicide myths (2.5–21.4%, p=0.001) and providing contact details for a suicide support service (32.8–71.4%, p=0.005). Conversely, there were largely no observable improvements in reporting by any individual Tamil-language newspaper.

**Conclusions:**

We observed substantial improvements in the reporting quality of some English-language newspapers, with minimal improvements in the quality of reporting in Tamil-language newspapers. The media guidelines in India are supporting the early phases of a culture shift on media reporting of suicide, yet they are just the start of the conversation. Strategies are required to engage and support vernacular language newspapers in India on their reporting of suicide, with media sector leadership as a core component.

**STRENGTHS AND LIMITATIONS OF THIS STUDY**

- We followed nine major newspapers at two time points, and our analyses were based on a large census sample of 1631 newspaper articles of suicides from 2016 and a random sample of 512 newspaper articles from 2023.
- We only examined newspaper reporting, and future studies might examine other forms of media.
- We only present data on the quality of reporting at two time points, 2016 and 2023. We would have ideally undertaken a form of time series analysis, with data from multiple time points, to track the trend over time.

## Background

 The epidemiology and sociocultural aspects of suicide vary substantially around the world,^1^ yet it is severely under-researched outside of a small number of high-income ‘western’ countries.^2^ Despite global suicide rates reducing 33% since 1990, suicide rates remain stubbornly high in South Asia, the location of four in ten global suicides, with a suicide rate 60% higher than the global average.^1 3^ The emerging interest in addressing suicide in South Asia is hampered by resource constraints, driving a clear imperative for low-cost interventions.

Suicide rates in India are among the highest in the world, with the most recent suicide death rate estimates ranging between 18 and 21 deaths per 100 000 population (compared with the global average of 11/100 000). This equates to an estimated 2 30 000–2 50 000 suicide deaths annually with far-reaching social, emotional and economic consequences.^3 4^ A public health approach to suicide prevention has gained momentum in India over the past decade,^5^ and the first national suicide prevention strategy was recently developed.^6 7^ The strategy takes a multisectoral approach and identifies the media sector as a key area for suicide prevention.

One of the few recommended suicide prevention strategies at the population level is responsible media reporting of suicides.^8^,^10^ Numerous studies have demonstrated that some media reports of suicide incidents can be a stimulus for imitation acts by vulnerable people.^11^,^13^ Evidence suggests that the imitation risk is exacerbated by sensational and graphic reporting practices, such as by publishing a detailed description of the suicide method, and when the coverage is of a celebrity suicide.^11 12^ Furthermore, media reports can unintentionally lead to the dissemination of suicide methods and behaviours^14^ and can be a source of misinformation through providing simplistic monocausal explanations for suicide. Importantly, research has also observed that suicide rates reduce following non-fictional and fictional media stories about people who have found ways to live with suicidal thoughts and who implemented alternative actions to suicidal behaviour; this has become known as ‘the Papageno effect’.^11 15 16^ Based on this evidence base, the WHO and the International Association for Suicide Prevention partnered to develop voluntary media guidelines, which recommend that public health specialists should engage with media professionals to limit irresponsible media coverage (eg, reports that sensationalise suicide) and to promote coverage that educates the public about suicide.^17^

The mass media market in India has observed exponential growth and diversification in the number of mass media outlets since the market was privatised in the late ‘90s.^18^ While other countries have observed a decline in print media, India has maintained steady annual growth in terms of publications and income.^19 20^ Younger populations are increasingly embracing news on their screens, and newspapers in India have adapted to have a strong digital presence. The print media industry is growing at 3.4% annually, with a 5% year-on-year growth in ad space.^20^ Competition is fierce to attract lucrative advertising revenue, and there is a high level of global commercial interests in the purchasing power of the rapidly expanding middle class in India.^21^ As in many countries, this has seen the rapid expansion of a ‘24/7’ breaking news culture. Alongside this trend, the diversity of cultures in India has seen strong demand for a wide array of local/regional news channels, catering to diverse languages and tastes.^18^ Of the 400 million copies of newspapers circulated daily, approximately 50% are in Hindi, and the remainder are in English and a wide range of regional vernacular languages.^20^

The manner by which the mass media communicates with the Indian public on the topic of suicide had until recently gone without scrutiny.^22^ Our own recent research found a very high frequency of graphic, explicit and simplistic media reporting of suicide in Tamil Nadu, India, predominantly undertaken by crime journalists.^23 24^ We observed that, on average, daily newspapers published one suicide article per day, with the majority being brief (ie, 10 sentences or less) incident reports. Potentially harmful reporting practices were common, such as providing a detailed description of the suicide method, while potentially helpful practices, such as providing contact details for suicide support services, were rare. Other studies from India have documented similar findings.^24^,^31^ While high-quality epidemiological evidence on copycat/imitation suicides in relation to media reports has remained elusive due to issues around the way suicide data are collated and disseminated in India, studies have examined proxy indicators for this phenomenon. For example, we documented that suicide-related media events in India, such as celebrity suicides, are associated with large increases in highly concerning suicide-related internet search queries (eg, ‘*how to hang yourself*’), with search queries mirroring the suicide method described in media coverage.^32^ We further documented through qualitative interviews that: media reporters on the crime beat work in close partnership with police to produce routine and simplified incident report-style coverage of suicide incidents; that suicide reports are used as ‘clickbait’ to generate audience interest; and that media professionals are largely receptive to voluntary media guidelines around suicide reporting, although with doubts around compliance, unless a systematic approach to dissemination and media engagement is undertaken, accompanied by initiatives to engage media professionals at the highest levels who can direct editorial practices.^33 34^

In late 2019, the Press Council of India adopted the WHO media guidelines, encouraging media professionals to follow this voluntary guidance.^35^ These guidelines were accompanied by Project Siren, a national-level media monitoring project based in western India, which focused on suicide reporting by English-language print and online media.^36^ However, there has been no systematic examination of the impact on media reporting. In 2016, we had undertaken a content analysis of the quality of newspaper reporting in the southern state of Tamil Nadu (population=72 million), which consistently has one of the highest suicide rates in India (25.9/100 000) equating to 19 834 recorded suicides in 2022 (ie, over 50 suicides per day).^37^ In Tamil Nadu, additional efforts to engage with media on this issue involved a local suicide prevention non-government organisation (NGO) (SNEHA) hosting a forum on media and suicide, the integration of training on the issue within a prominent college of journalism and research studies aimed at understanding the local dynamics around suicide reporting.^23 33 34 38^ In this study, we repeated the content analysis in 2023 to evaluate whether the characteristics and quality of newspaper reporting of suicides in Tamil Nadu had changed in alignment with the guidelines adopted by the Press Council of India.

## Methods

### Study design

The study involved using a repeat quantitative content analysis methodology, with data extracted from print newspaper reports in Tamil Nadu in 2016 and again in 2023. Quantitative content analysis is used to systematically quantify the presence of certain characteristics in media reports. We repeated the same content analysis methodology in both 2016 and 2023, to evaluate whether the characteristics and quality of newspaper reporting of suicides in Tamil Nadu had changed in alignment with the guidelines adopted by the Press Council of India. The adoption of the WHO media guidelines by the Press Council of India took place in 2019,^39^ allowing time for the absorption and implementation of guideline-supported media reporting. While a lot of newspaper content has now moved over to an online presence, this was not the case during our initial study in 2016. Hence, we have repeated the same approach and focused on print media reports only. Print media reports also allow examination of some additional characteristics, such as which page the article was published on. It also allows more certain identification of suicide-related stories, without relying on search terms for online news reports that may miss relevant articles that do not use specific key words.

### Sampling

In 2016, we undertook a content analysis study of articles reporting suicide-related news in nine of the 10 most highly read vernacular and English-language daily newspapers in Tamil Nadu over the 7-month period between 1 June and 31 December 2016. The findings and a detailed description of the methods are described elsewhere.^23^ The nine newspapers collectively had an estimated average daily readership of over 16 000 000 people in Tamil Nadu alone.^40^ Five of the nine newspapers were in the top 20 most circulated daily newspapers in the country,^41^ giving the findings relevance beyond Tamil Nadu.

To source the articles, three trained research assistants (psychologists) hand searched the hard copies of all 1926 (9 newspapers × 214 days) editions of the nine newspapers during the study period, allowing us to include several newspapers that did not have a strong online presence. Our search yielded 1681 suicide-related articles.

We included articles that primarily reported on specific instances of non-fictional suicide events, including deaths, non-fatal attempts or ideation/threats. We also included articles that primarily contained general commentary on the issue of suicide, including discussion of high-risk groups, research findings, prevention programmes or initiatives, raising awareness of suicide or commentary on any other aspect of suicide/suicide prevention. We excluded articles where suicide was only mentioned briefly (ie, <50% of the article) and articles with a focus on terrorist-related suicide bombings or euthanasia.

In 2023, we repeated this exercise and examined reporting in the same newspapers over the 6-month period between 1 July and 31 December 2023. To be able to detect a small change (Cohen's d=0.2) in reporting characteristics (eg, a drop from 43% to 39% in the percentage of articles reporting the suicide method in detail), we estimated that we needed a sample of 394 suicide articles (80% power; two-tailed t-test; 95% CI). In our prior study, we observed that newspapers published 0.9 suicide articles per day, on average. Based on this, we estimated that we needed to randomly select 60 days during the study period (July–Dec 2023; 184 days) in order to obtain 486 suicide articles, which would meet our sample size requirements. Our search for suicide articles on these 60 randomly selected days yielded 512 suicide articles for our analyses.

### Data extraction

In both 2016 and 2023, the same bilingual (English, Tamil) psychologist and researcher (MPsych, MPhil) extracted data from the suicide articles. First, descriptive information was extracted from each article, including: name of newspaper, section of newspaper, number of sentences, the primary focus of the article (ie, reporting on a suicide event vs a commentary-style article), the type of suicide event reported (ie, suicide death, attempt, ideation/threat) and whether or not the suicide event was connected to an instance of homicide-suicide or a suicide pact. Second, a quality assessment was undertaken to evaluate each article against the WHO suicide reporting guidelines.^17^ A comprehensive coding frame with operational definitions and examples for each item was designed to guide the coder in identifying a range of potentially harmful as well as helpful reporting characteristics, which we published with the results of our 2016 study.^23^ Each characteristic was coded as being either present (1) or absent (0).

We evaluated the inter-rater reliability of the quality assessment through a pilot study of 100 articles that were not part of the final sample, to ensure the main rater was producing reliable findings. Two independent coders used the coding frame to assess the quality of the pilot study articles. Cohen’s Kappa ranged from 0.84 to 1.0, with an average of 0.95, indicating strong inter-rater reliability.^42^ Additionally, throughout the study, meetings were regularly held to seek agreement in relation to any minor doubts that arose during the coding process.

### Statistical analysis

Our analyses were based on 1681 suicide articles from 2016 and 512 suicide articles from 2023. We used Stata V.16.0 for data analysis. We used frequencies, percentages and mean (95% CIs) to describe the data. Two-sample tests of proportions and independent sample t-tests on aggregate frequencies/proportions, along with 95% CIs, were used to assess whether there were statistically discernible (p<0.05) changes in the characteristics and quality of suicide articles between 2016 and 2023.

## Results

### Changes in the frequency and characteristics of reporting

Table 1 displays data on the volume of suicide articles in each newspaper in 2016 and 2023. Across all newspapers combined, there was a small yet statistically discernible increase in the average number of suicide articles per day per newspaper from 2016 (0.87) to 2023 (0.95). There was substantial variation across newspapers, with three newspapers (Daily Thanti, The Hindu, Deccan Chronicle) having a statistically discernible reduction in the frequency of suicide reporting and four newspapers (Dinakaran, Dinamalar, Malai Malar, Times of India) having a significant increase. In 2023, the three newspapers with the highest volume of suicide articles per day were all Tamil-language newspapers (Daily Thanti, Dinakaran, Dinamalar), which were followed by the English-language publication Times of India. Of the four newspapers with the lowest volume of suicide articles per day, three were English-language newspapers (The Hindu, Deccan Chronicle, New Indian Express), and one was a Tamil-language newspaper (Dinamani).

**Table 1:** Frequency and density of suicide articles in nine major newspapers in Tamil Nadu

Newspaper	2016			2023		
	n	%	Average number of suicide articles, per newspaper, per day*	n	%	Average number of suicide articles, per newspaper, per day†
Daily Thanthi (T)	498	29.6	2.33	111	21.7	1.85
Dinakaran (T)	160	9.5	0.75	99	19.3	1.65
Dinamalar (T)	220	13.1	1.03	77	15.0	1.28
The Hindu (E)	119	7.1	0.56	14	2.7	0.23
Dinamani (T)	139	8.3	0.65	25	4.9	0.42
Malai Malar (T)	115	6.8	0.54	53	10.4	0.88
Deccan Chronicle (E)	183	10.9	0.86	35	6.8	0.58
Times of India (E)	137	8.2	0.64	66	12.9	1.10
New Indian Express (E)	110	6.5	0.51	32	6.3	0.53
*Across all newspapers*	1681	100	0.87	512	100	0.95

Note: (T) signifies a Tamil language publication and (E) signifies an English language publication.

* For 2016, the average number of suicide articles per newspaper, per day, was calculated by dividing the total number of articles from each newspaper by the number of days (ie, 214 days) between June and December 2016. The average across all newspapers was calculated by dividing the total number of articles by the number of days (ie, 214 days) and then dividing by the number of newspapers (ie, 9).

† For 2023, the average number of suicide articles per newspaper, per day, was calculated by dividing the total number of articles from each newspaper by the number of days (ie, 60 days) randomly selected between July and December 2023. The average across all newspapers was calculated by dividing the total number of articles by the number of days (ie, 60 days) and then dividing by the number of newspapers (ie, 9).

We observed some small yet statistically discernible changes in the descriptive characteristics of suicide articles between 2016 and 2023 (see table 2). There was a small reduction (86.6–82.2%) in the proportion of articles published in the main section of the newspaper, with an increasing proportion of articles going into supplement sections. Suicide articles were typically longer in length in 2023, with the average length rising from a mean of 11.6 sentences in 2016 to a mean of 13.4 sentences in 2023. Relatedly, we saw a small reduction (96.0% to 93.4%) in the proportion of articles that had a primary focus on reporting on suicidal behaviour and a commensurate increase (4.0–6.6%) in articles that were primarily providing a commentary on the topic of suicide. Among those articles reporting on a suicide event, we observed an increase (77.4–85.1%) in the proportion focused on suicide deaths and a reduction (21.8–17.4%) in the proportion focused on non-fatal suicide attempts; articles focused on someone’s experience of suicidal ideation reduced from 2.0% to 0.2%. Among articles focusing on suicidal ideation, only one article in 2016 and none in 2023 focused on a positive story of someone who drew on strengths and resources to overcome a suicidal crisis. There was no significant change in the reasonably high proportion (relative to the rate at which these events occur in the official statistics) of suicide articles that were covering homicide-suicide events or suicide pact events.

**Table 2:** Descriptive characteristics of suicide articles in Tamil Nadu in 2016 and 2023

Characteristics	2016 (n=1681)		2023 (n=512)		Difference(2023–2016)	P value
Section of newspaper	N	% (95% CI)	N	% (95% CI)	% (95% CI)	
Main section	1455	86.6 (84.9, 88.2)	421	82.2 (78.9, 85.5)	−4.3 (-0.6, 8.0)	0.0147
Supplement	226	13.4 (11.8, 15.1)	90	17.6 (14.3, 20.9)	4.1 (0.5 7.8)	0.0197
Number of sentences						
<11 sentences	916	54.5 (52.1, 56.9)	241	47.1 (42.7, 51.4)	−7.4 (-2.5, 12.4)	0.0032
11–20 sentences	585	34.8 (32.5, 37.1)	204	39.8 (35.6, 44.1)	5 (0.2, 9.9)	0.0374
>20 sentences	179	10.6 (9.2, 12.1)	67	13.1 (10.2,16.0)	2.4 (0.8, 5.7)	0.126
Mean number of sentences	11.6	11.6 (11.2, 12.0)	13.4	13.4 (12.5,14.4)	1.8 (0.9, 2.7)	<0.001
Average number of articles per newspaper, per day	0.87	(0.84, 0.89)	0.95	(0.90, 0.99)	−0.08 (-0.02, 0.14)	0.005
Primary focus of article						
Report of suicide event(s)	1613	96 (95, 96.9)	478	93.4 (91.2, 95.5)	−2.6 (-0.2, 4.9)	0.0146
Commentary on an aspect of suicide	68	4.0 (3.1, 5.0)	34	6.6 (4.5, 8.8)	2.6 (0.2, 4.9)	0.0146
Type of suicide events covered (among those who reported suicide events)						
Suicide death events	1249	77.4 (75.4, 79.5)	407	85.1 (82, 88.3)	7.7 (3.9, 11.5)	<0.001
Non-fatal suicide attempt events	351	21.8 (19.7, 23.8)	83	17.4 (14, 20.8)	−4.4 (-0.4, 8.3)	0.0374
Suicidal ideation/threat events	33	2.0 (1.4, 2.7)	1	0.2 (-0.2, 0.6)	−1.8 (−1.0, 2.6)	0.0053
Suicide event is situated within a story of homicide-suicide or suicide pact (among event articles)						
Homicide-suicide event	157	9.7 (8.3, 11.2)	57	11.9 (9.0, 14.8)	2.2 (-5.4,1.1)	0.1651
Suicide pact event	209	13 (11.3, 14.6)	54	11.3 (8.5, 14.1)	−1.7 (-1.6,4.9)	0.3364

### Changes in the quality of reporting

We also observed small yet statistically discernible improvements in the quality of reporting between 2016 and 2023 (see table 3). There was a significant reduction (4.9–1.8%) in the proportion of articles placed on the front page and a reduction (92.7–86.5%) in reporting of the suicide method. There was also a trend towards a reduction (43.1–38.9%) in detailed descriptions of the suicide method (ie, at least two specific details provided as to how the method was enacted), although the p value was marginal (p=0.092). Several helpful reporting characteristics had increased with statistical significance, including: recognition of the link between suicide and poor mental health (7.6–10.5%), recognition of the link between suicide and alcohol and other substance dependence/use (4.4–8.2%), the provision of population-level data related to suicide (2.6–4.9%), mentions of suicide prevention support services/programmes (3.6–11.7%) and the provision of contact details for a suicide support service (2.5–8.8%).

**Table 3:** Changes in the quality media reporting of suicide in Tamil Nadu between 2016 and 2023

Potentially harmful characteristics	2016(n=1681)		2023 (n=512)		Difference(2023–2016)	P value
	n	% (95% CI)	n	% (95% CI)	% (95% CI)	
Highly prominent placement						
Front page	83	4.9 (3.9, 6)	9	1.8 (0.6, 2.9)	−3.18 (−4.72, 1.64)	0.002
First three pages	283	16.8 (15, 18.6)	98	19.1 (15.7, 22.5)	2.31 (-1.54, 6.15)	<0.001
Suicidal act						
Suicide method reported	1559	92.7 (91.5, 94)	443	86.5 (83.6, 89.5)	−6.22 (−9.43, 3.01)	<0.001
Detailed account of method (ie, at least two specific details about how the method was implemented)	724	43.1 (40.7, 45.4)	199	38.9 (34.6, 43.1)	−4.2 (-9.04, 0.64)	0.092
Public site named as location of a suicide death/attempt	138	8.2 (6.9, 9.5)	51	10 (7.4, 12.6)	1.75 (-1.16, 4.66)	0.216
Causes of suicidality						
Negative life event(s) related to suicide reported (eg, debt)	1366	81.3 (79.4, 83.1)	404	78.9 (75.4, 82.4)	−2.35 (-6.35, 1.64)	0.237
Monocausal explanation for suicidality	897	53.4 (51, 55.7)	362	70.7 (66.8, 74.6)	17.34 (12.73, 21.95)	<0.001
Details from suicide note reported	160	9.5 (8.1, 10.9)	72	14.1 (11.1, 17.1)	4.54 (1.22, 7.87)	0.003
Headlines						
‘Suicide’ in the headline	1219	72.5 (70.4, 74.7)	381	74.4 (70.6, 78.2)	1.9 (-2.44, 6.24)	0.397
Suicide method in the headline	669	39.8 (37.5, 42.1)	199	38.9 (34.6, 43.1)	−0.93 (-5.76, 3.9)	0.706
Life event(s) in the headline	661	39.3 (37, 41.7)	206	40.2 (36, 44.5)	0.91 (-3.93, 5.76)	0.712
Consideration for bereaved persons						
Interview with bereaved persons	46	2.7 (2, 3.5)	8	1.6 (0.5, 2.6)	−1.17 (-2.5, 0.15)	0.133
Photos						
An accompanying photo	470	28 (25.8, 30.1)	181	35.4 (31.2, 39.5)	7.39 (2.73, 12.06)	0.001
Photo of a suicidal person	362	21.5 (19.6, 23.5)	149	29.1 (25.2, 33)	7.57 (3.17, 11.96)	<0.001
Potentially helpful characteristics						
Causes of suicidality						
Recognises link with poor mental health	128	7.6 (6.3, 8.9)	57	11.1 (8.4, 13.8)	3.52 (0.51, 6.52)	0.012
Recognises link with substance dependence/use	74	4.4 (3.4, 5.4)	42	8.2 (5.8, 10.6)	3.8 (1.23, 6.37)	<0.001
Dispels common suicide myths						
Dispels the myths that there are no preceding warning signs and/or that there is nothing you can do to prevent suicide	34	2 (1.3, 2.7)	9	1.8 (0.6, 2.9)	−0.26 (-1.59, 1.06)	0.705
Draws on health experts, research and data to inform the public						
Expert opinion from a mental health professional	21	1.2 (0.7, 1.8)	10	2 (0.8, 3.2)	0.7 (-0.61, 2.01)	0.238
Population level data/statistics related to suicide	44	2.6 (1.9, 3.4)	25	4.9 (3, 6.7)	2.27 (0.25, 4.28)	0.010
Raises awareness of prevention services						
Mentions a suicide prevention programme/support service	60	3.6 (2.7, 4.5)	60	11.7 (8.9, 14.5)	8.15 (5.23, 11.07)	<0.001
Provides contact details for a suicide support service	42	2.5 (1.8, 3.2)	45	8.8 (6.3, 11.2)	6.29 (3.73, 8.85)	<0.001

We also observed some statistically discernible increases in the proportion of articles containing potentially harmful reporting characteristics. There was a significant increase in the proportion of articles: providing simple monocausal explanations for suicidal behaviour (53.4–70.7%), publishing details from a suicide note (9.5–14.1%), providing an accompanying photograph (28.0–35.4%) and providing a photo of a suicidal person (21.5–29.1%). There was no statistically significant change in the use of potentially harmful reporting characteristics in the headlines of suicide articles. The proportion of headlines that contained the word suicide (72.5–74.4%), the suicide method (39.8–38.9%) and a life event purported to be the driver of the suicide (39.3–40.2%), remained unchanged.

### Variation by newspaper language and by newspaper

The quality of reporting varied by newspaper language and within specific newspapers (see online supplemental tables 1, 2). Most of the improvements observed in reporting quality outlined earlier were driven by changes in articles published in English language newspapers. Among English-language newspapers, we also observed a statistically discernible decrease in the proportion of articles providing a detailed account of the suicide method (43.2–31.3%) and a decrease in the use of the word ‘suicide’ in headlines (51.0–36.1%).

While our study was statistically underpowered to analyse within newspaper changes, it was evident that profound changes in the quality of reporting by two particular newspapers (The Hindu and New Indian Express) were mainly responsible for the improvements in reporting quality that we documented above. The most substantial improvements in reporting were observed for the English-language newspaper, The Hindu, where a reduction in overall reporting of suicide was matched by an increase in reporting quality. At The Hindu, there were statistically discernible reductions in suicide articles: on the front page (7.6–0.0%), mentioning the suicide method (85.7–14.3%), providing a detailed account of the suicide method (42.9–14.3%), using the word suicide in headlines (53.8–28.6%), mentioning the suicide method in headlines (17.7–0.0%) and providing the photo of a suicidal person (10.9– 0.0%). There were also statistically discernible increases in suicide articles: dispelling suicide myths (2.5–21.4%), providing expert opinions from mental health professionals (1.7–14.3%), providing population-level data on suicide (2.5–64.3%) and providing contact details for a suicide support service (32.8–71.4%).

Similarly, at the English-language newspaper New Indian Express, there were statistically discernible reductions in the proportions of suicide articles: on the front page (16.4–3.3%), mentioning the suicide method (90.0–31.3%), providing a detailed account of the suicide method (43.6–6.3%), naming the public site of a suicide death/attempt (8.2–0.0%), using the word suicide in headlines (61.8–28.1%), mentioning the suicide method in headlines (29.1–0.0%) and providing the photo of a suicidal person (20.9–12.5%). Further, there was a statistically significant increase in suicide articles mentioning suicide prevention programmes (2.7–78.1%) and providing contact details for a suicide support service (0.0–75.0%). Encouragingly, at the English-language newspaper Times of India, there were also signs of some early progress with a statistically significant increase in suicide articles mentioning suicide prevention programmes (3.7–19.7%) and providing contact details for a suicide support service (0.7–13.6%).

## Discussion

Responsible media reporting of suicide is advocated as an effective population-level suicide prevention strategy,^3 8^ aimed at reducing imitation suicide deaths, improving suicide-related attitudes and preventative practices and showing due consideration to bereaved persons.^3 43^ Our prior study had found that, in 2016, there was a daily diet of short, explicit, repetitive, simplistic and potentially harmful suicide-related news that was served up to readers of popular daily newspapers in Tamil Nadu, India, with a low level of adherence to WHO suicide reporting guidelines. We examined whether the characteristics and quality of reporting had changed since the Press Council of India adopted the WHO media guidelines in 2019. We identified some encouraging signs, with small improvements on a number of quality indicators. However, there were no improvements in several quality indicators, and reporting had worsened on some quality indicators. Analyses at the newspaper level showed that the small improvements that were observed were mainly driven by quite profound improvements in the quality of reporting by two English-language newspapers, which represent something of a proof of concept that voluntary guidelines can support changes in media practices in India.

The dominant pattern of newspaper reporting of suicide in India is for crime reporters to produce short incident-based reports (ie, who, what, how, when, where) on a ‘newsworthy’ selection of suicide deaths and attempts,^23 33 44^ and our results indicate this is still the case. Nonetheless, we observed a reduction in the proportion of suicide articles that are being published on the front page of newspapers and fewer are reporting the suicide method, both of which are important changes to reduce the prominence of the articles and the graphic detail around the suicide method.

It was also encouraging to observe a trend towards a longer length of suicide articles and a related increase in the number of articles taking a commentary-style approach rather than only focusing on specific suicide events. Providing greater length to the articles appears to have facilitated a complimentary increase in the use of population-level statistics, greater recognition of poor mental health and alcohol/substance use as contributors to suicide risk and mentions of suicide prevention programmes/services. The public health approach to suicide prevention has gained recent traction in India, with the development of the first national suicide prevention strategy^6^ and new national helpline services,^45^ coupled with an increased awareness of suicide during the most intense periods of the COVID-19 pandemic and in the aftermath of a very high-profile celebrity suicide in 2020.^32 46^ It is encouraging to see that, in alignment with this, newspaper coverage in Tamil Nadu appears to be making some progress towards providing more meaningful and educative coverage.

Importantly, analyses at the newspaper level revealed that these improvements can be largely attributed to profound changes in the reporting approach by two particular newspapers. The Hindu and New Indian Express both demonstrated widespread changes across a number of quality indicators. This is a really encouraging sign and may provide a template for others to follow. Our qualitative interviews with media professionals at The Hindu back in 2018 indicated that they were already starting to think about making changes,^34^ prior to the Press Council adopting media guidelines and prior to Project Siren. They reported having developed a greater awareness of the issue through building a relationship with a suicide prevention NGO and helpline (SNEHA) in Chennai. The relationship was a long-standing one with multiple engagement opportunities. For example, the SNEHA suicide prevention helpline had initially recruited volunteers for their helpline through advertisements provided through The Hindu at no cost; the Chairman of the media company was invited to an event held by the NGO, with sessions on the topic of media reporting of suicide, and a decision was taken to develop an in-house policy brief.^38^ What is evident from our new study is that a concerted effort was clearly made to improve their coverage of suicide news. These changes may have taken place regardless of the new national media guidelines, but the presence of guidelines can only assist in providing a helpful strategic direction for the industry. Nonetheless, the story signifies the power of local advocacy and the importance of building relationships with those at the highest levels within media companies.

It is critical to note that both of these two newspapers primarily publish in English, which we believe is highly relevant for a few reasons. First, the WHO media guidelines are written in English and it may be that engaging with English-language media may represent an easier ‘early win’ in efforts to engage media on this issue in India. Second, English-language media outlets often have a presence across several states of the country and may be more likely to be exposed to conversations around this issue that occur in the other major international cities of India through initiatives like Project Siren. Third, the growth in the newspaper industry in India has been driven heavily by the growth of vernacular language print media and preferences for local news in local languages.^18 20^ Notably, in our recent 2023 data, Tamil language newspapers had the highest volume of suicide articles per issue, and there were few signs of changes in the quality of reporting in these newspapers. It will be critical for media engagement efforts on this issue to develop a strategy for systematic engagement with vernacular press across India. Localised engagement, rather than national-level efforts, might be more effective in working with vernacular press, and this may need to include examples of how best-practice suicide articles can be written in different languages.

A critical body of evidence has emerged around the importance of ‘Papageno’ narratives that cover the stories of people who experience suicidal thoughts and find ways to cope and survive the period of crisis. A recent meta-analysis found that media narratives of hope and recovery from suicidal crises have a beneficial effect on suicidal ideation in individuals with some vulnerability.^16^ Unfortunately, in our study, we observed that this type of reporting remains absent from media coverage of suicide in India. Suicidal ideation without an accompanying suicide attempt is the most common experience of suicidality in the population, yet it is rarely considered a newsworthy ‘event’ in the Indian context (nor may other places in the world) where it is especially deaths that make news.^33^ It also appears highly unlikely that positive stories of surviving suicide crises will emerge through the current dominance of suicide coverage by crime reporters in India. This is an important area for further research and media engagement in India, and it is noteworthy that the Press Council of India has thus far focused their communications more on what media should not do (eg, avoid reporting on suicide methods, etc), rather than what media should do (eg, stories of people surviving suicide crises).^35^ This is consistent with press councils elsewhere in the world, where the emphasis is on what should be avoided in reporting, with a reluctance to be telling media what should be reported. A compounding issue to navigate is the high level of stigma associated with suicidality in India, where public disclosures of suicidality and mental health challenges may negatively impact individuals and their families.^47^ Nonetheless, emerging research from India indicates that direct contact with people with lived experience of mental health challenges can reduce stigmatising attitudes,^48^,^50^ as has been documented elsewhere in the world.^51^ Further research should examine the effects of suicide disclosures in mass and social media formats in the Indian context, looking at effects on suicidal ideation among people with some vulnerability to suicide, as well as effects on other outcomes like stigmatising attitudes and intended help-seeking.

Overall, our findings are somewhat comparable to observations in other countries after the introduction of media guidelines. Similar incremental improvements were observed in Hong Kong after the dissemination of a manual on suicide reporting based on the WHO guidelines.^52^ Larger improvements were observed in Canada and Australia^53 54^; however, the populations and media sectors are smaller in these countries than in India, there are fewer languages to contend with and media guidelines have been accompanied by more systematic engagement with the media sector. Interestingly, similar to the Australian experience,^54^ the volume of suicide articles in India has increased slightly since the introduction of the media guidelines, perhaps indicative of growing interest in the issue of suicide. From a public health perspective, daily reporting on suicide deaths/attempts in every edition of a newspaper is not likely to be helpful for the population, and this could be moderated with a focus on a more moderate number of high-quality reports containing protective reporting elements. Similar to the Canadian experience,^53^ there was a moderate increase in simplistic monocausal explanations for suicide in India. Suicide is a complex and multifaceted phenomenon, and there can be a temptation in media reporting to focus on recent ‘triggers’ for a suicide event, particularly given short media articles are not necessarily conducive to more complex conversations around suicide. Future media engagement activities might explore appropriate examples of how journalists can best handle discussing recent trigger events while conveying a more nuanced understanding of the complexity of suicide.

The move by the Press Council of India to adopt and disseminate the WHO media guidelines has been a really encouraging first step and represents the beginning of a longer journey of culture change. A review of the effectiveness of media guidelines for suicide reporting indicates that approaches centred on collaboration and media ownership are likely to be most effective and need to be coupled with active dissemination.^55^ In settings like Australia and Austria, the development and adoption of media guidelines have led to robust ongoing contact between media professionals and mental health experts, and ultimately, programmes targeting suicide reporting have come to be led by media professionals.^56^ The journey on this issue is in its infancy in India, and it would be a mistake to presume that the issue is now a closed case simply because the Press Council of India has adopted media guidelines for suicide reporting. The story of change (see above) at The Hindu highlights the importance of building productive personal relationships with media companies. Moving forward in India, there is a clear need for a robust implementation strategy to address the implementation challenges associated with responsible media reporting of suicide. Additionally, a strategy is required for engaging with the vernacular press. In our study, four of the nine newspapers were in the English language, yet only 18% of the population in Tamil Nadu is literate in English. Furthermore, the WHO media guidelines are written in English, the PCI dissemination of the media guidelines was also in English and early initiatives like Project Siren have also focused on English-language press. Given the clear need for engagement with vernacular press, dissemination of media guidance is needed in vernacular languages.

The study has several limitations that are worth noting. First, we only looked at newspapers, and with a larger budget, it would have been desirable to examine reports by other forms of mass media. Additionally, further studies might like to examine online media, which may allow the collection of data like readership statistics as well as an assessment of any accompanying video content. Second, we only looked at newspapers from Tamil Nadu; however, five of the nine newspapers were in the top 20 most-read newspapers in the country, and the English-language newspapers in particular have a broad readership across the country. Third, we were statistically underpowered to examine changes in reporting within specific newspapers. Given that the improvements in reporting we observed could largely be attributed to substantial changes in two particular newspapers, we recommend future research to allow for a sufficient sample size for within-newspaper analyses. Fourth, we only present data on the quality of reporting at two time points, 2016 and 2023. We would have ideally undertaken a form of time series analysis, with multiple data from multiple time points, to track the trend over time. However, we were not able to access the daily newspaper issues retrospectively and thus needed to rely on prospective data collection in 2023. Fifth, we do not know how newspapers in India engaged or not with the new media guidelines, and qualitative studies may be helpful to unpack how these guidelines were received and any barriers and facilitators that impacted efforts to change. A case study documenting how the changes took place at the two newspapers where profound improvements have happened would be helpful as a study of positive deviance. Sixth, while we were interested to see if the quality of media reporting had improved since the adoption of media guidelines in India, our pre-post study design is unable to account for other potential influential events that might have had an effect on reporting quality. For example, prior research has highlighted some changes in the profile of suicides during the COVID-19 pandemic,^57^ and the dissemination of the National Suicide Prevention Strategy in 2022 may have had a positive effect. The remarkable similarity in our findings between 2016 and 2023, aside from improvements in two English-language media outlets, suggests these events were unlikely to be a major driver of a change in reporting quality. Finally, we would ideally have looked at the effects of media coverage of suicide news on changes in suicide rates in Tamil Nadu. Given we only observed small changes in media reporting, it would be unrealistic to expect to observe any changes in suicide rates. Furthermore, data on suicides in India are collated by the National Crime Records Bureau, which unfortunately suffers from issues with systemic underenumeration and is an unreliable source of data for this purpose.^58^

### Conclusion

We observed some encouraging signs that newspapers are beginning to display improvements in suicide reporting in Tamil Nadu, India, although with small effects that appear to be isolated to English-language newspapers. We recommend that a more systematic approach be undertaken to improving media reporting of suicide in India, with strategic initiatives to engage the vernacular press.

## Supplementary material

10.1136/bmjopen-2024-092652 online supplemental table 1

## Data Availability

Data are available upon reasonable request. All data relevant to the study are included in the article or uploaded as supplementary information.
