# Supplementary material for: Progress towards prevention of suicide in India by improving print media reporting of suicide news: a repeat content analysis study in Tamil Nadu
Source: BMJ Open. 2025 May 16;15(5):e092652. doi: 10.1136/bmjopen-2024-092652 (PMC12086940; doi:10.1136/bmjopen-2024-092652)
Supplement: Supplementary file 2 [file bmjopen-2024-092652.reviewer_comments.pdf]

PEER REVIEW HISTORY

BMJ Open publishes all reviews undertaken for accepted manuscripts. Reviewers are asked to complete a checklist review form and are provided with free text boxes to elaborate on their assessment. These free text comments are reproduced below.

ARTICLE DETAILS

Title (Provisional)

Progress towards prevention of suicide in India by improving print media reporting of suicide news: a repeat content analysis study in Tamil Nadu

Authors

Armstrong, Gregory; Jayaseelan, Mala; Niederkrotenthaler, Thomas; Cherian, Anish; Menon, Vikas; Arya, Vikas; Haregu, Tilahun; Vijayakumar , Lakshmi

---

VERSION 1 - REVIEW

**Reviewer**	**1**
**Name**	**Werdin, Sophia**
**Affiliation**	**Harvard T H Chan School of Public Health, Environmental Health**
**Date**	**27-Sep-2024**
**COI**	**None**

I thank the editor for the opportunity to review this interesting manuscript. The authors have examined the association between the adoption of WHO media reporting guidelines by the Press Council of India and changes in the amount and quality of newspaper articles related to suicide in Tamil Nadu, India. They conducted a before-and-after comparison using qualitative content analysis. Below, I offer several suggestions that I believe could enhance the manuscript in terms of clarity, comprehensiveness, and impact.

1. I recommend explicitly stating in the title that the study focused exclusively on print media (and on Tamil Nadu, India).

2. The abstract currently highlights only the positive changes found in the study. I suggest also including the negative changes both in the abstract and in the first paragraph of the discussion to give a more balanced and comprehensive overview of the study's findings.

3. At the end of the abstract and conclusion, it would be beneficial to propose concrete strategies for improving adherence to the media guidelines, particularly for vernacular newspapers.

4. It would be helpful to include information on which language/s the WHO guidelines were made available in and how they were disseminated. Although this is briefly mentioned in the discussion, it is a key aspect that could have influenced the study's results and should be introduced earlier in the paper, i.e. in the introduction.

5. Since the introduction and discussion mention that an Indian suicide prevention strategy was developed recently, it would be helpful to specify when exactly this occurred and how it may have impacted media reporting.

6. Furthermore, elaborating on the role of the Press Council in India in the introduction would be valuable. What does it mean for newspapers when the Press Council makes a recommendation or adopts a guideline? Is it simply advisory, or is there a degree of obligation or pressure on media outlets to comply?

7. The introduction would benefit from some general aspects of suicide in India, as cultural factors strongly influence how suicidal behavior is experienced, expressed, perceived, and framed, as well as how prevention is approached. In this context, it would also be worth discussing the limitations of adapting media guidelines across different cultural contexts (in the discussion).

8. Either "Southeast Asia" or "South Asia" should be used consistently in the introduction.

9. A few terms could be refined: for example, the phrase "dynamics of suicide" could be more specific, and "emerging appetite" could be rephrased as "emerging interest".

10. In the objective, it should be made clear that the study is not a nationwide analysis but specifically focuses on Tamil Nadu (or southern India).

11. Given the differentiation of newspapers by languages, it would be helpful to provide some context regarding the proportion of English-speaking inhabitants in this area to give an indication of the potential audience reached by the English-language newspapers (in comparison to vernacular newspapers). Additionally, a brief characterization of the newspapers included in the study (e.g., in terms of content, target audience, ownership) would provide further context on the diversity/comparability of the media analyzed.

12. The discussion could also consider the potential mediating effects of the Project Siren, which is mentioned in the introduction. Since this project focuses on suicide reporting by English-language media, it would be useful to discuss its potential influence on the outcomes of the study.

13. Moreover, the manuscript should consider whether any critical events (e.g., COVID-19, national suicide prevention strategy introduction) occurred before or during the study periods, which could have affected media reporting and the study's results.

14. Finally, the reference list appears somewhat imbalanced, as a significant portion of the cited articles are authored by the authors of this manuscript. A greater diversity of scientific evidence would strengthen the credibility of the literature review.

**Reviewer**	**2**
**Name**	**Groves, Samantha**
**Affiliation**	**University of Oxford Department of Psychiatry**
**Date**	**03-Feb-2025**
**COI**	**None**

Research Summary and Overall Impression

Many thanks for the opportunity to review this article. For context, I am currently a DPhil Psychiatry student. In 2023, I completed a study using content analysis to examine news reporting of suicide related to nurses.

This is an important and interesting study given the high incidence of suicide, and the prominence and growth of news agencies in India. The researchers have used an established method and appropriate framework to analyse the data. This study gives useful initial insights related to progress since adoption of media guidelines on responsible reporting of suicide in 2019, as well as useful implications for collaboration with Tamil-language newspapers.

I hope that the comments below are useful.

Best wishes,

Samantha Groves

General Point

Print vs Online Articles: whilst reading the article I wondered about online news articles published during the study period, particularly given the current strong digital presence as mentioned. These articles may include additional features to assess against WHO guidelines e.g., the presence of videos or photo galleries, alongside ‘clickable’ links to support. These articles can also give interesting information about the reach and reception of articles e.g., statistics shown about how many times the article has been shared, and comments shared on articles. As the current study repeated methods used in 2016, I realise that this may not have been feasible, but it may be useful to provide a rationale within the methods section about why online articles were not assessed, and exploration as a future research implication may be worthwhile in the discussion section.

Comments by Section

Abstract:

• It would be helpful to state in the abstract whether the newspapers reviewed were in print or online format, alongside the period studied e.g., 6-month window in 2023.

• As the results from the statistical analysis (with p values) are displayed in the abstract results section, it would be helpful to state what analysis was conducted to generate these results within the abstract methods section (e.g., two-sample tests of proportions and independent sample t-tests).

Background: Overall, I found the introduction to be well written, and the context of both suicide and print media in India, alongside the rationale for the study are clear. A few suggestions related to readability:

• Page 5, lines 8-9: in the first paragraph, there appears to be two references presented using brackets, i.e., (1), whereas the rest of the paper uses superscript i.e., 1. The references appear to be correct so just reformatting is needed.

• Page 5, lines 51-54: I found the sentence beginning "competition is fierce" quite difficult to understand, rewording to "Competition is fierce to attract lucrative advertising revenue, with global commercial interest in the purchasing power of the rapidly expanding middle class in India" may improve readability.

• Page 6, line 19: This qualitative work seems very interesting and an important way to explain the context this current study is conducted in. However, I found this section slightly difficult to read. I would be interested to see some further clarity about who the interviews were conducted with, and more of a commentary of the findings rather than reporting the results in list form.

• Page 6, line 40- it would be helpful to define the acronym NGO, particularly as it is used a few times in article.

Methods: A note this is a very impressive amount of work to carry out. I know it takes a huge effort to source and analyse these news articles.

• Although this is a repetition of previous methods used, for readers new to the topic it would be helpful to provide a rationale for using content analysis and briefly what this entails.

• As mentioned above a rationale for focusing on print articles would be helpful.

• When describing the data extracting author, it would be useful to state which languages are spoken by the researcher where it states they are bilingual (e.g., English and Tamil?).

• It would be useful to state what the authors defined as a statistically discernible result within the data analysis paragraph.

Results

When reading the results I found myself looking for the p values within the text, although you do helpfully state where the results are statistically discernible. The paper may benefit from either signposting the readers to the tables to view the associated p values within the Data Analysis section of the Methods or stating these values alongside the reported results.

Discussion

• As discussed above, the exploration of online articles among these newspapers may be a future research implication warranted for discussion.

• The discussion may benefit from some additional attention related to the increases in potentially harmful content e.g., publication of the details of suicide notes, and future implications related to these.

Tables

• In Table 2 the title for confidence intervals uses rounded brackets % (95% CI) whereas for Table 3 square brackets are used at the top of the column % [95% CI].

• Within the tables there is some variance as to spacing of the statistics with confidence intervals. I found where spaces are used the values are easier to read. E.g., 11.6 (11.2, 12.0) is clearer than 11.6(11.2,12.0).

• In Table 3, I cannot see explanation of what the * next to the p value title denotes.

VERSION 1 - AUTHOR RESPONSE

Reviewer 1

'In the abstract, I recommend reporting the suicide rate instead of the absolute number of annual suicides in India, as this would facilitate comparisons with other countries. Additionally, the mention of "over 200,000 suicides" in the abstract, alongside "230,000–250,000" in the introduction, appears somewhat imprecise.'

We have now updated the abstract as suggested.

Reviewer 2

'A couple of minor issues I spotted can be corrected during proofing:
- "things had also worsened on some quality indicators". Possibly replace the word 'things' for further clarity. E.g., reporting had worsened on some quality indicators.
- "our pre-post study design is unable to account for other potential influence of other events on reporting quality" Possibly needs rewording to something like 'other potential influential events which affected reporting quality' to enhance clarity.'

We have edited the text as suggested.

VERSION 2 - REVIEW

**Reviewer**	**1**
**Name**	**Werdin, Sophia**

**Affiliation**	**Harvard T H Chan School of Public Health, Environmental Health**
**Date**	**02-Apr-2025**
**COI**	

I thank the authors for thoughtfully considering my comments in the revised version of the paper. I believe the revisions have improved the clarity and transparency of the article.

I only have one minor suggestion:

In the abstract, I recommend reporting the suicide rate instead of the absolute number of annual suicides in India, as this would facilitate comparisons with other countries. Additionally, the mention of “over 200,000 suicides” in the abstract, alongside “230,000–250,000” in the introduction, appears somewhat imprecise.

**Reviewer**	**2**
**Name**	**Groves, Samantha**
**Affiliation**	**University of Oxford Department of Psychiatry**
**Date**	**17-Apr-2025**
**COI**	

Many thanks to the authors for their thoughtful responses. I believe the article is suitable for accepting. A couple of minor issues I spotted can be corrected during proofing:

"things had also worsened on some quality indicators". Possibly replace the word 'things' for further clarity. E.g., reporting had worsened on some quality indicators.

"our pre-post study design is unable to account for other potential influence of other events on reporting quality" Possibly needs rewording to something like 'other potential influential events which affected reporting quality' to enhance clarity.

VERSION 2 - AUTHOR RESPONSE

Reviewer 1

‘I thank the editor for the opportunity to review this interesting manuscript. The authors have examined the association between the adoption of WHO media reporting guidelines by the Press Council of India and changes in the amount and quality of newspaper articles related to suicide in Tamil Nadu, India. They conducted a before-and-after comparison using qualitative content analysis. Below, I offer several suggestions that I believe could enhance the manuscript in terms of clarity, comprehensiveness, and impact.’

We thank the reviewer for these encouraging opening remarks.

1. *‘I recommend explicitly stating in the title that the study focused exclusively on print media (and on Tamil Nadu, India).’*

We have adapted the title as suggested.

2. *‘The abstract currently highlights only the positive changes found in the study. I suggest also including the negative changes both in the abstract and in the first paragraph of the discussion to give a more balanced and comprehensive overview of the study’s findings.’*

We have updated the abstract and first paragraph of the discussion as recommended.

3. *‘At the end of the abstract and conclusion, it would be beneficial to propose concrete strategies for improving adherence to the media guidelines, particularly for vernacular newspapers.’*

The abstract already signposts that: ‘The media guidelines in India are supporting the early phases of a culture shift on media reporting of suicide, yet they are just the start of the conversation. Strategies are required to engage and support vernacular language newspapers in India on their reporting of suicide, with media sector leadership as a core component.’

We believe this is sufficient for an abstract. We have added some additional text to the second last paragraph of the discussion section that highlights the need for a robust implementation strategy, and the need for close engagement with vernacular press. This paragraph already highlights, based on prior experience elsewhere, the importance of close partnership between mental health experts and media professionals, and the need for media leadership. We believe this is sufficient as the focus of the article is on the quality of the media reporting. We plan to publish a detailed road map of strategies to address media reporting of suicide elsewhere.

4. *‘It would be helpful to include information on which language/s the WHO guidelines were made available in and how they were disseminated. Although this is briefly mentioned in the discussion, it is a key aspect that could have influenced the study’s results and should be introduced earlier in the paper, i.e. in the introduction.’*

This is an important observation, and we fully agree with the reviewer. The WHO guidelines are published in English. The Press Council of India dissemination and endorsement of media guidelines was also in English. We have added a statement around this in the last paragraph of the discussion section, which highlights the need for guidelines to be disseminated in vernacular languages.

5. *‘Since the introduction and discussion mention that an Indian suicide prevention strategy was developed recently, it would be helpful to specify when exactly this occurred and how it may have impacted media reporting’*

The strategy was published in 2022. We have updated the references to add a direct reference to the strategy. We are not suggesting the national suicide prevention strategy will have had time to impact the media reporting. The strategy is high level, and isn’t for media professionals, specifically. It simply identifies media reporting of suicide as one area to focus on in the future. Our paper was focused on evaluating whether the characteristics and quality of newspaper reporting of suicides had changed in alignment with the guidelines adopted by the Press Council of India in 2019.

6. *‘Furthermore, elaborating on the role of the Press Council in India in the introduction would be valuable. What does it mean for newspapers when the Press Council makes a recommendation or adopts a guideline? Is it simply advisory, or is there a degree of obligation or pressure on media outlets to comply*?’

The reviewer makes an important observation. We have now indicated in the background section that the guidelines dissemination by the Press Council are voluntary.

7. *‘The introduction would benefit from some general aspects of suicide in India, as cultural factors strongly influence how suicidal behavior is experienced, expressed, perceived, and framed, as well as how prevention is approached. In this context, it would also be worth discussing the limitations of adapting media guidelines across different cultural contexts (in the discussion).’*

The background section is already 6 paragraphs long. We believe it is beyond the scope of this particular paper to start detailing the socio-cultural aspects of suicide in India. The paper is focused on media reporting, and we are aiming to keep the focus fairly tight in that regard.

8. *‘Either "Southeast Asia" or "South Asia" should be used consistently in the introduction.’*

Thank you, we believe we have no corrected this.

9. *‘A few terms could be refined: for example, the phrase "dynamics of suicide" could be more specific, and "emerging appetite" could be rephrased as "emerging interest".’*

We have made the necessary corrections.

10. *‘In the objective, it should be made clear that the study is not a nationwide analysis but specifically focuses on Tamil Nadu (or southern India).’*

We have made the necessary correction.

11. *‘Given the differentiation of newspapers by languages, it would be helpful to provide some context regarding the proportion of English-speaking inhabitants in this area to give an indication of the potential audience reached by the English-language newspapers (in comparison to vernacular newspapers). Additionally, a brief characterization of the newspapers included in the study (e.g., in terms of content, target audience, ownership) would provide further context on the diversity/comparability of the media analyzed.’*

In the last paragraph of the discussion, we have elaborated further on the need to engage the vernacular press. In doing so, we have added information that 18% of the population in Tamil Nadu is literate in English.

We don’t have access to other information about these publications. There are media tracking/analysis private companies who can provide this information at a cost, but we don’t have funds to acquire this.

12. *‘The discussion could also consider the potential mediating effects of the Project Siren, which is mentioned in the introduction. Since this project focuses on suicide reporting by English-language media, it would be useful to discuss its potential influence on the outcomes of the study.’*

We have acknowledged the potential positive influence of Project Siren on the English language press in the discussion section. Nonetheless, the qualitative interviews we undertook in a prior study emphasised that changes were taking place at these English-language media houses as a result more so because of local advocacy and engagement efforts in Chennai.

13. *‘Moreover, the manuscript should consider whether any critical events (e.g., COVID-19, national suicide prevention strategy introduction) occurred before or during the study periods, which could have affected media reporting and the study’s results.’*

We have acknowledged this in the limitations section.

14. *‘Finally, the reference list appears somewhat imbalanced, as a significant portion of the cited articles are authored by the authors of this manuscript. A greater diversity of scientific evidence would strengthen the credibility of the literature review.’*

We do sincerely appreciate this comment, and we take it seriously and we have reflected on this. Our team has undertaken several high-quality studies on this topic in recent years, each study building on the other, hence there is a sequence of references from the work published by the authors on this paper. Additionally, some of our authors are international leaders in this field and have published some of the highest quality and most highly cited pieces of evidence from different locations. We don’t propose to remove these references.

Nonetheless, we have identified and added 10 new references to our paper to broaden the literature base. If the reviewer would like to highlight any further important references that add new important contextual information that ought to be included, we will consider them.

Reviewer: 2
‘This is an important and interesting study given the high incidence of suicide, and the prominence and growth of news agencies in India. The researchers have used an established method and appropriate framework to analyse the data. This study gives useful initial insights related to progress since adoption of media guidelines on responsible reporting of suicide in 2019, as well as useful implications for collaboration with Tamil-language newspapers. I hope that the comments below are useful.’

We thank the reviewer for these encouraging opening remarks.

1. *‘Print vs Online Articles: whilst reading the article I wondered about online news articles published during the study period, particularly given the current strong digital presence as mentioned. These articles may include additional features to assess against WHO guidelines e.g., the presence of videos or photo galleries, alongside ‘clickable’ links to support. These articles can also give interesting information about the reach and reception of articles e.g., statistics shown about how many times the article has been shared, and comments shared on articles. As the current study repeated methods used in 2016, I realise that this may not have been feasible, but it may be useful to provide a rationale within the methods section about why online articles were not assessed, and exploration as a future research implication may be worthwhile in the discussion section.’*

We have now flagged this in the limitations as a suggestion for future research.

2. *‘It would be helpful to state in the abstract whether the newspapers reviewed were in print or online format, alongside the period studied e.g., 6-month window in 2023.’*

We have updated the title of the manuscript to refer to print media, to avoid any confusion. We have also stated this in the abstract.

3. ‘*As the results from the statistical analysis (with p values) are displayed in the abstract results section, it would be helpful to state what analysis was conducted to generate these*

results within the abstract methods section (e.g., two-sample tests of proportions and independent sample t-tests).'

This is mentioned in the 'design' section of the abstract.

4. '*Background: Overall, I found the introduction to be well written, and the context of both suicide and print media in India, alongside the rationale for the study are clear.'*

We thank the reviewer for these encouraging remarks.

5. '*Page 5, lines 8-9: in the first paragraph, there appears to be two references presented using brackets, i.e., (1), whereas the rest of the paper uses superscript i.e., 1. The references appear to be correct so just reformatting is needed.'*

We have corrected this error.

6. '*Page 5, lines 51-54: I found the sentence beginning "competition is fierce" quite difficult to understand, rewording to "Competition is fierce to attract lucrative advertising revenue, with global commercial interest in the purchasing power of the rapidly expanding middle class in India" may improve readability.'*

We have attempted to improve the readability of the sentence.

7. *'Page 6, line 19: This qualitative work seems very interesting and an important way to explain the context this current study is conducted in. However, I found this section slightly difficult to read. I would be interested to see some further clarity about who the interviews were conducted with, and more of a commentary of the findings rather than reporting the results in list form.'*

We thank you for your interest in this work. We too believe it is really interesting and important work. We had to keep our discussion of this brief in the background section, as the background was already 6 paragraphs long. We highlighted what we felt were the most pertinent aspects for this current study:

- That media reporters on the crime beat work in close partnership with police to produce routine and simplified incident report-style coverage of suicide incidents
- That suicide reports are used as "clickbait" to generate audience interest
- That media professionals are largely receptive to voluntary media guidelines around suicide reporting, although with doubts around compliance unless a systematic approach to dissemination and media engagement is undertaken, accompanied by initiatives to engage media professionals at the highest levels who can direct editorial practices

8. *'Page 6, line 40- it would be helpful to define the acronym NGO, particularly as it is used a few times in article.'*

We have corrected this.

9. *‘Methods: A note this is a very impressive amount of work to carry out. I know it takes a huge effort to source and analyse these news articles. Although this is a repetition of previous methods used, for readers new to the topic it would be helpful to provide a rationale for using content analysis and briefly what this entails.’*

Thank you for acknowledging the exhaustive efforts that went into this study, it is a lot of work! We have added some brief words related to content analysis in the study design section to improve the clarity of the study design. We feel the rest of the methods are fairly succinct and clear for a scientific journal article.

10. *‘As mentioned above a rationale for focusing on print articles would be helpful.’*

We have added this to the study design section of the methods.

11. *‘When describing the data extracting author, it would be useful to state which languages are spoken by the researcher where it states they are bilingual (e.g., English and Tamil?).’*

We have now added this.

12. *‘It would be useful to state what the authors defined as a statistically discernible result within the data analysis paragraph.’*

We have now added this.

13. *‘When reading the results I found myself looking for the p values within the text, although you do helpfully state where the results are statistically discernible. The paper may benefit from either signposting the readers to the tables to view the associated p values within the Data Analysis section of the Methods or stating these values alongside the reported results.’*

We have now signposted our definition of statistically discernible ($p<0.05$) in the data analysis section of the methods. As you rightly state, the tables also have the specific information on the actual p-value.

14. ‘*As discussed above, the exploration of online articles among these newspapers may be a future research implication warranted for discussion.’*

We have now added this, as mentioned above.

15. ‘*The discussion may benefit from some additional attention related to the increases in potentially harmful content e.g., publication of the details of suicide notes, and future implications related to these.’*

We have now noted in the discussion and the abstract that some indicators had worsened.

16. 'In Table 2 the title for confidence intervals uses rounded brackets % (95% CI) whereas for Table 3 square brackets are used at the top of the column % [95% CI].'

We have now corrected this.

17. '*Within the tables there is some variance as to spacing of the statistics with confidence intervals. I found where spaces are used the values are easier to read. E.g., 11.6 (11.2, 12.0) is clearer than 11.6(11.2,12.0).'*

We will leave this for the journal formatting team to decide based on their standard processes.

*18. 'In Table 3, I cannot see explanation of what the * next to the p value title denotes.'*

Thank you for spotting this error. We have removed the asterisk as it is redundant. The data analysis section indicates what tests were used.
